# BAC-FISH refutes report of an 8p22–8p23.1 inversion or duplication in 8 patients with Kabuki syndrome

**DOI:** 10.1186/1471-2350-7-46

**Published:** 2006-05-18

**Authors:** Kendra W Kimberley, Colleen A Morris, Holly H Hobart

**Affiliations:** 1 Department of Pediatrics/Division of Genetics, University of Nevada School of Medicine, Las Vegas, NV, USA

## Abstract

**Background:**

Kabuki syndrome is a multiple congenital anomaly/mental retardation syndrome. The syndrome is characterized by varying degrees of mental retardation, postnatal growth retardation, distinct facial characteristics resembling the Kabuki actor's make-up, cleft or high-arched palate, brachydactyly, scoliosis, and persistence of finger pads. The multiple organ involvement suggests that this is a contiguous gene syndrome but no chromosomal anomalies have been isolated as an etiology. Recent studies have focused on possible duplications in the 8p22–8p23.1 region but no consensus has been reached.

**Methods:**

We used bacterial artificial chromosome-fluorescent *in-situ *hybridization (BAC-FISH) and G-band analysis to study eight patients with Kabuki syndrome.

**Results:**

Metaphase analysis revealed no deletions or duplications with any of the BAC probes. Interphase studies of the Kabuki patients yielded no evidence of inversions when using three-color FISH across the region. These results agree with other research groups' findings but disagree with the findings of Milunsky and Huang.

**Conclusion:**

It seems likely that Kabuki syndrome is not a contiguous gene syndrome of the 8p region studied.

## Background

Kabuki syndrome [OMIM 147920] is a multiple congenital anomaly/mental retardation syndrome of unknown etiology. The syndrome was first described independently by Niikawa *et. al*. and Kuroki *et. al*. in 1981 [1,2]. There is no definitive laboratory diagnostic test for Kabuki syndrome, thus diagnosis is made based on phenotypic presentation and by ruling out other known syndromes. The typical characteristics of Kabuki syndrome are: varying degrees of mental retardation, postnatal growth retardation, characteristic dysmorphic facial features, congenital heart disease, brachydactyly, scoliosis, and persistence of prominent finger pads.

The dysmorphic facies are characterized by long palpebral fissures with eversion of the lateral part of the lower eyelids, arched eyebrows, broad, depressed nasal tip, prominent ears, cleft or high-arched palate, and dental/occlusal abnormalities. Individuals with Kabuki syndrome have relative strengths in verbal and reasoning abilities and weakness in visuospatial construction; adaptive behavior is characterized by good social interactions and communication but poor motor and personal living skills [3]. The multiple organ involvement suggests that Kabuki syndrome is a contiguous gene syndrome but no etiology has been determined. There have been several cases of Kabuki syndrome with various chromosomal anomalies but none have had any cytogenetic anomaly in common [4-6]. Milunsky and Huang [7] recently reported finding a duplication of 8p22–8p23.1 in six unrelated patients with Kabuki syndrome. They also reported finding a submicroscopic inversion in all six patients and in two mothers of Kabuki syndrome children. However, studies conducted by Miyake *et. al*. [5], Schoumans *et. al*. [6], Miyake *et. al*. [8], Hoffman *et. al*. [9], Engelen *et. al*. [10], Sanlaville *et. al*. [11], and Turner *et. al*. [12] failed to find evidence of the duplications reported by Milunsky and Huang. We investigated eight patients with Kabuki syndrome and six normal controls to determine if either an inversion or a duplication in 8p22–8p23.1 exists.

## Methods

### Clinical evaluation

Eight individuals with Kabuki syndrome identified through the University of Nevada School of Medicine Genetics Program consented to participate in the study. Medical records were reviewed and physical examinations were completed. The diagnosis was based upon the presence of the typical pattern of malformations and dysmorphic features reviewed by Matsumoto and Niikawa [13].

### Cytogenetics

Blood was collected from patients diagnosed with Kabuki syndrome, their parents, and normal controls according to approved Institutional Review Board procedures. Blood lymphocytes were cultured using standard cytogenetic techniques with thymidine used for synchronization. The lymphocytes were dropped onto clean, wet slides to obtain well spread metaphase chromosomes and interphase nuclei. The slides were allowed to air dry and were then baked for 1 ½ hours at 90°C. Analysis of metaphase chromosomes occurred by using GTG-banding standard techniques.

### Fluorescent *in-situ *hybridization (FISH)

Ten bacterial artificial chromosome (BAC) clones that both Milunsky and Huang [7] and Miyake *et. al*. [8] reportedly used (RP11-11P7, RP11-140K14, RP11-122N11, RP11-235F10, RP11-112G9, RP11-252K12, RP11-31B7, RP11-92C1, RP11-23H1, and RP11-141K9) were obtained from the Children's Hospital Oakland Research Institute (Oakland, CA) human genomic library. The BACs were grown and the DNA harvested using standard methods. The ends of the BAC DNA insert were sequenced at the Nevada Genomics Center at the University of Nevada, Reno, NV. The sequences were compared to published NCBI genomic sequences using the BLAST program to confirm the location of the BAC DNA on the human genome.

The BAC DNA was then labeled separately in both Spectrum Red and Spectrum Green using a Nick Translation Kit (Vysis, Downers Grove, IL). The labeled DNA was co-precipitated with Human Hybloc (Cot-1) DNA (Applied Genetics Laboratories, Melborne, FL) and tested on normal control slides to confirm labeling success.

To detect inversions, three-color FISH was performed by applying probes labeled in Spectrum Red and Spectrum Green. The third color, yellow, was achieved by mixing equal parts of red and green labeled probe. Prepared slides of both Kabuki patients and normal controls were serially dehydrated in ethanol and allowed to air dry. Labeled BAC probe was applied. The slides were coverslipped and sealed with rubber cement. Co-denaturation of probe and target DNA occurred at 77.5°C for 6 minutes followed by hybridization at 37°C overnight. The coverslips were removed and the slides washed in 50% formamide/2X SSC at 50°C for 15 minutes followed by a wash in 2X SSC at 37°C for 8 minutes to remove any mismatched probe. The slides were air dried, counterstained with DAPI/Antifade, coverslipped, and sealed. The slides were then examined by fluorescence microscopy and scored for probe number and probe order.

## Results and discussion

All eight individuals met the clinical criteria for a diagnosis of Kabuki syndrome. They all had the Kabuki syndrome distinctive facial features (long palpebral fissures with everted lateral lower lids, arched eyebrows, short nasal septum, and prominent ears) and the typical physical findings (hypotonia, prominent fingertip pads, and joint laxity). The additional clinical features are summarized in Table 1.

**Table 1:** Kabuki syndrome clinical features. The clinical features of the eight Kabuki patients included in this study.

Patient	1	2	3	4	5	6	7	8
Age	8 y	10.7 y	9 y	26 y	11.5 y	9 y	11.5 y	13 mo
Sex	M	M	M	F	F	F	F	F
Age of Dx	6 y	1 mo	9 mo	8 mo	11.5 y	9 y	5 y	7 mo
Development								

Single Words	24 mo	5 y	24 mo	19 mo	30 mo	12 mo	36 mo	No
Walking	16 mo	18 mo	22 mo	16 mo	3.5 y	12 mo	30 mo	No
Hearing Loss	No	No	No	No	Yes	No	No	No

Growth%								

L	40%	30%	<3%	50%	<3%	25%	<5%	75%
W	60%	80%	<3%	80%	<3%	98%	70%	25%
OFC	30%	98%	<3%	5%	2%	50%	20%	<2%

Anomalies								

Palate					Cleft palate			Lip pit
Cardiac *	BAV	DAR	ASD	AS	BAV			Mitral insufficiency
Renal				Pelvic kidney				
Premature thelarche						+	+	
Scoliosis							+	
Other *	Crypt.			GER, Ant. anus	GER, Strabismus, CDH, Nystagmus	Strabismus	Seizure disorder, Hemivertebra	GER, IgA and IgG deficiency

* AS, aortic stenosis; BAV, bicuspid aortic valve; ASD, atrial septal defect; DAR, dilated aortic root; GER, gastroesophageal reflux; Crypt., cryptorchidism; CDH, congenital dysplasia of hip; Ant. anus, anterior placement of the anus.

### Cytogenetics

Five or more cells were analyzed for each patient following U.S. clinical cytogenetic standards. No cytogenetic abnormalities were noted by either G-band analysis of at least the 500-band level resolution or by targeted high resolution analysis of 8pter of the Kabuki patients.

### BAC-FISH

#### Metaphase FISH

##### Normal controls

The BAC probes were tested against normal controls to confirm the probe location and signal number. Except BAC RP11-122N11, all BAC probes showed two equally strong signals on all metaphases and interphase nuclei. BAC RP11-122N11 showed four or five signals on all control metaphases and interphases and was rejected for use in this study. Interestingly, this BAC was reported by Milunsky and Huang as being inverted in their Kabuki patients [7]. Hollox *et. al*. [14] and Sanlaville *et. al*. [11] have mapped this BAC to the middle of the β-defensin-gene cluster, a gene cluster that is polymorphic for copy number, corresponding to gene SPAG11 at 8p23.1. This BAC also matched with multiple loci on multiple chromosomes when the sequenced ends were compared to known human genomic sequences using the NCBI BLAST program.

##### Kabuki patients

The remaining nine BAC probes were tested on eight Kabuki syndrome probands. All probes yielded two equally strong signals on both metaphase chromosomes and interphase nuclei indicating no deletion or duplication of these probes. This supports the recent findings of three genome-wide array CGH screenings that did not reveal any pathological findings in Kabuki patients, indicating that microduplications or microdeletions are less likely to be the cause of Kabuki syndrome [5,6,9].

#### Inversion FISH

**Figure 1:**
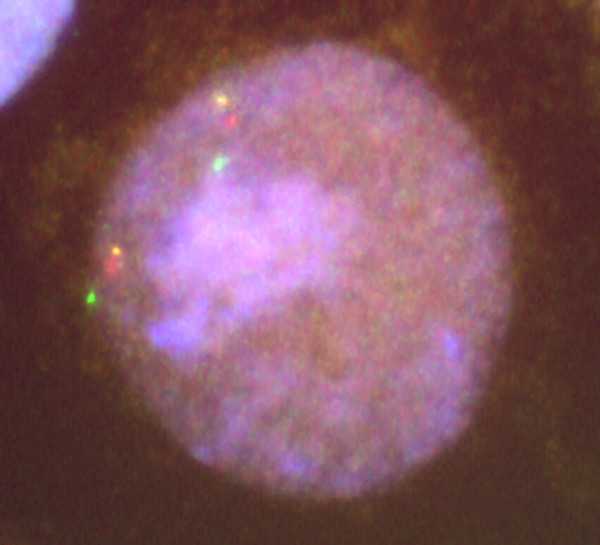
**Inversion FISH**. Image of three-color FISH used to detect an inversion in the region of a previously reported submicroscopic inversion. Probe order: PR11-11P7 (yellow), RP11-140K14 (red), and RP11-235F10 (green).

Three-color FISH was performed on interphase nuclei of normal controls to confirm the order of the BAC probes across the region. The order obtained agreed with the NCBI published sequence. Three-color FISH was then performed on interphase nuclei of all eight Kabuki probands. Forty-five interphase nuclei were scored on each patient to determine the probe order. A nucleus was considered scorable as long as the signals were in a relatively straight line and all three signals could clearly be seen. Milunsky and Huang reported a submicroscopic inversion between BACs RP11-122N11 and RP11-235F10 [7]. Since BAC RP11-122N11 was not used in this study due to the multiple numbers of signals seen in the normal controls, the BACs flanking RP11-122N11 and RP11-235F10 in sequence order were used for detecting an inversion. No inversions were seen between RP11-235F10 and RP11-140K14 (figure 1) or RP11-235F10 and RP11-112G9 (not pictured). No other inversions were detected across the region.

## Conclusion

To date, we have been unable to find evidence of any inversions, duplications, or deletions in the 8p22–8p23.1 region as reported by Milunsky and Huang [7]. Our results are consistent with those reported by Miyake *et. al*. [5], Schoumans *et. al*. [6], Miyake *et. al*. [8], Hoffman *et. al*. [9], Engelen *et. al*. [10], Sanlaville *et. al*. [11], and Turner *et. al*. [12]. The discrepancy that exists among the groups could be attributed to two possible scenarios: 1) the patients studied by Milunsky and Huang represent either a variant of Kabuki syndrome or a different syndrome of similar phenotypic characteristics (phenocopy), or 2) the reported duplication represents a normal variant in the 8p region as this region is known to undergo genetic rearrangement due to olfactory receptor-gene clusters causing unequal crossovers [6,11]; none of these genetic rearrangements have been associated with any clinical pathology. Further studies, possibly with collaboration between the groups, are required to determine why the discrepancies in the research results exist. However, it now seems less likely that Kabuki syndrome is caused by a contiguous gene rearrangement than by a mutation in a regulatory gene.

## Competing interests

The authors declare that they have no competing interests.

## Authors' contributions

KK participated in creating the experimental design, harvesting the BAC DNA, the sequence alignment, the FISH studies, and writing the draft of the manuscript. CM performed the clinical diagnosis of Kabuki syndrome patients. HH helped with the experimental design, culturing the BAC clones, and reading the karyotypes.

All authors read and approved the final manuscript.

## Pre-publication history

The pre-publication history for this paper can be accessed here:


